# Complete Genome Sequence of Bacillus velezensis KOF112, an Antifungal Endophytic Isolate from Shoot Xylem of the Indigenous Japanese Wine Grape *Vitis* sp. cv. Koshu

**DOI:** 10.1128/MRA.00422-21

**Published:** 2021-07-15

**Authors:** Kazuhiro Hamaoka, Shunji Suzuki

**Affiliations:** a Laboratory of Fruit Genetic Engineering, The Institute of Enology and Viticulture, University of Yamanashi, Kofu, Yamanashi, Japan; University of Maryland School of Medicine

## Abstract

*Bacillus* species are the most well-studied biological control agents and produce a large variety of antibiotics that exert antifungal activity against phytopathogens and induce defense responses in plants. Here, we introduce the genome sequence of Bacillus velezensis KOF112, an antifungal endophytic isolate from the shoot xylem of a wine grape.

## ANNOUNCEMENT

The mechanisms of plant disease suppression by *Bacillu*s species include antibiotic production, nutrient competition, and plant-mediated resistance induction (1, 2). We explored antifungal endophytic bacteria from grapevines. The surfaces of shoots of *Vitis* sp. cv. Koshu, a wine grape indigenous to Japan, were sterilized with sodium hypochlorite. Bark and epidermal tissue were peeled off from the shoots using a sterilized knife. Xylem was shaved using a sterilized grater and shaken into phosphate buffer (pH 7.4). After removing the xylem by filtration, the filtrate was incubated on soybean casein digest (SCD) plates at 25°C for 3 days. From 247 bacteria on the plates, *B. velezensis* KOF112 was obtained. To investigate the mechanisms of KOF112 antagonistic activities toward phytopathogenic fungi, genome sequencing of KOF112 was performed.

Genomic DNA was extracted from a 1-day culture of KOF112 in SCD medium at 25°C using a DNeasy PowerSoil kit (Qiagen). For short-read sequencing, a DNA library was prepared using an MGIEasy DNA Adapters-96 (plate) kit and an MGIEasy FS DNA library prep set (MGI Tech). Circularized DNA and DNA nanoballs were prepared using an MGIEasy circularization kit (MGI Tech) and a DNBSEQ-G400RS high-throughput sequencing kit (MGI Tech), respectively. DNBSEQ 2 × 200-bp paired-end sequencing was performed using a DNBSEQ-G400 sequencer (MGI Tech). After removing adapter sequences using Cutadapt v. 2.7, approximately 3.5 million read pairs (1.05 Gbp) were sampled from the sequence using SeqKit v. 0.11.0. Low-quality reads showing quality values less than 20 were removed using Sickle v. 1.33, and reads shorter than 127 and their paired reads were omitted. Finally, 6,581,706 high-quality paired-end reads were obtained. The average length of the short reads was 200 bp. For long-read sequencing using the GridION Nanopore sequencing platform by Oxford Nanopore Technologies (ONT), genomic DNA was barcoded using a native barcoding expansion kit (EXP-NBD114; ONT). A library was prepared using a ligation sequence kit (SQK-LSK109; ONT). After loading the library onto R9.4.1 flow cells and running it on a GridION device, base-calling and barcode segmentation for the raw sequences were performed using Guppy v. 4.0.11+f1071ce. Adapter sequences were removed using Porechop v. 0.2.3. Short reads (1,000 bp or less) were removed using Filtlong v. 0.2.0. Finally, 207,586 high-quality paired-end reads were obtained. The average length of the long reads was 3,877 bp. The high-quality short-read and long-read sequences were assembled using Unicycler v. 0.4.7 with default settings. The hybrid assembly generated two circular contigs with a coverage of 915×. The assembled contig graph was confirmed using Bandage v. 0.8.1, and the integrity of the assembled genomic data was confirmed using CheckM v. 1.1.2.

The genome includes two contigs with a size of 3,916,789 bp (circular genome) or 13,003 bp (plasmid). The *N*_50_ contig size and the longest contig size are the same (3,916,789 bp). The GC content is 46.5%. CheckM v. 1.1.2 indicated no contamination and 99.79% completeness of the genome. Annotation using DFAST v. 1.4.0 predicted 3,746 coding sequences, 27 rRNA genes, and 86 tRNA genes.

### Data availability.

This project was deposited in DDBJ/ENA/GenBank under accession numbers PRJDB11468 (BioProject), SAMD00293740 (BioSample), AP024603 (genome), AP024604 (plasmid), and DRA011804 (raw sequencing reads).
